# The activities of a dietitian-led gastroenterology clinic using extended scope of practice

**DOI:** 10.1186/s12913-016-1845-0

**Published:** 2016-10-21

**Authors:** Dominique Ryan, Fiona Pelly, Elizabeth Purcell

**Affiliations:** 1 Faculty of Science, Health, Education and Engineering, University of the Sunshine Coast, 90 Sippy Downs Dr, Sippy Downs, 4556 QLD Australia; 2 Faculty of Science, Health, Education and Engineering, University of the Sunshine Coast, Locked Bag 4, Maroochydore, 4558 QLD Australia; 3 Bundaberg Base Hospital, Bourbong Street, Bundaberg, 4670 QLD Australia

**Keywords:** Extended scope of practice, Dietetics, Quantitative

## Abstract

**Background:**

Extending the scope of practice of allied health professionals has been a strategy adopted in the United Kingdom to address issues within the health system.

Australia’s health system is currently undermined by similar issues, heightening government interest in adopting the extended scope health care model. The aim of the current study was to describe the activities and outcomes of a dietitian-led gastroenterology clinic which operated under an extended scope of practice model in an outpatient gastroenterology department at a tertiary hospital in regional Queensland, Australia, and to assess patient satisfaction with the initiative.

**Methods:**

A descriptive, cross-sectional case series undertaken over 50 clinics involving 82 category 2 and 3 patients with suspected/confirmed coeliac disease or inflammatory bowel disease; low haemoglobin; gastroesophageal reflux disease, or; malnutrition. Data was analysed using Microsoft Excel 2010, and presented as descriptive statistics.

**Results:**

Sixty out of 82 selected patients (median age 51 years) attended an initial appointment with the dietitian. Twenty-four review appointments were attended. Average waiting period for an initial appointment was 148 days (range 31–308 days). A total of 149 management strategies were provided, and 94 (63 %) of these involved the dietitian utilising extended scope of practice. The dietitian managed 47 (78 %) patients without need for gastroenterologist referral, and 25 (42 %) were discharged after dietetic management.

Patients reported high levels of satisfaction with the clinic.

**Conclusions:**

Seventy-eight percent of category 2 and 3 patients referred to the gastroenterologist could be managed exclusively in the dietitian-led clinic. This extended scope model of care could potentially benefit the efficiency and acceptability of Australia’s public health system.

## Background

The current Australian public health system is characterised by increased health care costs, physician shortages and prolonged patient waiting periods [1]. This has heightened interest in developing new health care models in order to maximise the efficiency of the health system [1, 2]. One alternative health care model which has proven effective in the United Kingdom (UK) is extended scope of practice for allied health practitioners [3]. Extended scope of practice can be defined by those who work in non-traditional positions, expanding on their customary roles in terms of diagnostics, management and consultation [3]. Allied health practitioners who engage in an extended scope of practice are commonly referred to as extended scope practitioners (ESPs) [3]. The evidence surrounding the benefits of ESPs suggests that they may be associated with many positive outcomes for stakeholders. These include decreased waiting times and costs, correct patient diagnosis, enhanced patient health outcomes, and increased satisfaction levels of patients, other practitioners and ESPs themselves [4–8]. To date, much of the focus has been on physiotherapists as ESPs. Nevertheless, ESP roles are reported in other allied health professions such as speech and language pathology, occupational therapy, paramedics and dietetics [6–8]. However, less is known about the implications of these roles for other allied health professions, particularly in the Australian context.

The ‘*Extended Scopes of Practice Program’* was developed by the Australian Government as part of the ‘*National Health Workforce Innovation and Reform Strategic Framework for Action 2011–2015*’ [1]. This program involved expanding the scope of particular allied health roles—specifically physiotherapists, nurses and paramedics - in four sub-projects to ascertain effects on recruitment, retention, productivity, accessibility, efficiency and effectiveness within the health care system [1]. Evaluation of these projects has led to the initial phases of development of an ‘*Extended Scope of Practice Toolkit’* to support the replication of similar ESP positions at a national level [9].

Within Australia, each state/territory is responsible for the provision of health services. More specifically on a state based level, the Queensland Government recognises the need to make changes to improve Queensland’s health services, being one of the many groups interested in the extended scope of practice model [2]. In 2013 the Queensland Ministerial Taskforce on Health Practitioner Expanded Scope of Practice was developed to identify ways in which health practitioners could expand their scope of practice in order to improve.

Queensland’s health services and the level of patient care in a cost effective manner [2]. A key recommendation from the Ministerial Taskforce was that further research be conducted into the outcomes of allied health practitioner extended scope of practice models in the Queensland context [2].

In Australia, dietitians are trained to provide nutrition information and prescribe dietary treatments to improve the health of individuals, communities and populations. However, there is little engagement through training or career progression in extended scope of practice. In the UK and America, a range of ESP positions exist for dietitians; including roles in stroke management, gastroenterology and outpatient enteral feeding [10–12]. Although a variety of extended scope of practice roles for dietitians are identified in the literature, few studies have focused on evaluating these roles [10, 12–22]. Nevertheless, the limited evidence suggests that extended scope roles for dietitians provide similar positive outcomes to those evidenced by the initiation of other allied health ESP roles. The only existing example of an ESP role for dietitians within Australia which has been the focus of a research investigation involved a dietitian working in a transdisciplinary model with speech pathologists [13]. Within this role, the dietitian undertook extended roles in dysphagia screening and intervention, and the extended scope model of care resulted in reducing the time for dietetic referral and assessment [13].

Given that the profession of dietetics is continuing to develop in the changing healthcare landscape, extending the scope of dietitians may be a possible means of developing career pathways and leadership in specific areas of practice.

The focus of the current study was a dietitian-led gastroenterology clinic operating under an extended scope of practice model at a tertiary hospital in regional Queensland, Australia. Gastrointestinal (GI) disease states commonly involve medical nutrition therapy from a dietitian. However, in this case, the dietitian acted as the first practitioner of contact; triaging patients to tests and providing nutritional advice where appropriate. Patients who could not be managed exclusively by the dietitian progressed to a consultation with the gastroenterologist. As limited Australian data exists on extended scope in dietetic practice, we aimed to describe the process, patient outcomes and patient satisfaction with the clinic.

## Methods

This study describes the activities of the dietitian-led gastroenterology clinic and outcomes of patients who attended the clinic during a specific time period from 01 November, 2013 to 31 March, 2015.

The site for this study was an outpatient gastroenterology department located at a tertiary hospital in regional Queensland, Australia. Data was collected between 01 November, 2013 and 31 March, 2015 from clinic sessions with both new and review patients.

The study involved a sole dietitian practicing with extended scope. The dietitian submitted an application to request credentialing and approved extended scope of clinical practice for the dietitian-led gastroenterology clinic to the local hospital service’s Credentialing and Defining Scope of Clinical Practice Committee. Approval was gained from both the Committee and the local Director of Medical Services for a 3 year period. To sustain credentialing rights, the dietitian was required to; a) practice only within the work areas and service provisions requested on the application; b) maintain professional development and competence in the areas of extended practice.

The dietitian acted as a first practitioner of contact; providing screening, assessment and interventions for patients who met the inclusion criteria. The dietitian was credentialed to undertake extended scope tasks such as requesting pathology tests, colonoscopies, and scans. Patients were referred to the gastroenterologist as required based on established clinical guidelines. The patient management strategies developed by the dietitian were approved by the gastroenterologist prior to implementation.

Participants who attended the clinic during the study period and who met the inclusion criteria for the study were invited to participate. This included Category 2 patients (defined as having a condition that could potentially require more complex care if treatment is delayed) and Category 3 patients (having a condition that is unlikely to deteriorate quickly or require more complex care if treatment is delayed). Category 1 patients (those who have a condition that will likely deteriorate quickly if care is delayed) where excluded from the study. Inclusion criteria included patients who presented with a condition that could be potentially managed by a dietitian. That is, they were referred to the outpatient clinic with suspected/confirmed coeliac disease or inflammatory bowel disease, undiagnosed low haemoglobin, gastroesophageal reflux disease or malnutrition. Eligible patients were initially advised via a mailed letter that their referral had been reviewed and was appropriate for the dietitian-led clinic. Details regarding the purpose of the clinic were also provided in the letter.

Participation in the clinic was voluntary, and patients were informed of their right to wait to see the gastroenterologist if preferred.

Data on patient demographics (age, condition/symptom, number of days waiting to attend the clinic after referral, patient attendance/non-attendance and self-reported reasons for nonattendance) and outcomes after attending the clinic were collected. Patients were categorised depending on the site of their initial condition/symptom (upper GI, lower GI, other). Decisions regarding patient management were classified as within the dietitian’s scope of practice (referred to gastroenterologist, dietary intervention, scheduled for review) or outside of the dietitians scope of practice (laboratory test/medical imaging/endoscopy requested, escalated up to category 1, suggested medication alterations, discharged).

Data on patients’ self-reported levels of satisfaction with the clinic was obtained using an anonymous survey developed for the purpose of the study by one of the researchers (an experienced dietitian) based on previous experience measuring patient satisfaction for other quality activities conducted in the hospital setting. Specifically, questions aimed to measure patients’ satisfaction with their entire clinic journey from the initial referral to the clinic, to consultation with the dietitian and treatment outcome. The survey included negatively phrased questions to reduce acquiescence response bias, and was piloted with other health professionals, including another expert research dietitian, for content validity and revised where necessary. Patients were mailed the survey after attending the clinic to minimise social desirability bias, which may have been be introduced into the study design if surveys were completed while researchers were present [23]. Patients were asked to rate their level of satisfaction with aspects of the dietitian-led clinic on a five-point Likert scale from “strongly disagree” to “strongly agree”. Specifically, the survey assessed patient satisfaction with five key components of the clinic, as outlined in Table 1.

**Table 1:** Satisfaction survey

Key component assessed	Survey statement
Information provision	- My appointment letter gave me enough information on this clinic - The purpose of the clinic was not explained to me during my appointment
Waiting periods	- I waited too long for my appointment after I accepted - On the day of my appointment I was seen on time - This clinic allowed me to access health care earlier than I had expected
Dietitian’s advice and care	- The advice I received was easy to understand - I am confident in the advice given to me by the Dietitian - I feel my concerns were listened to
Management outcomes	- I am satisfied with the outcome of this consultation
Clinic experience	- Thinking about your entire clinic experience how satisfied are you?

Data was analysed using Microsoft Excel 2010, and presented descriptively. Pearson's chi-squared test was used to test for association between patient groups (i.e. lower GI, upper GI or other) and patients who received outcomes which involved the dietitian utilising an extended scope of practice.

## Results

Eighty-two patients (median age 51 years) met the inclusion criteria and were eligible to attend an initial appointment. Between November 1, 2013 and March 31, 2015, 60 patients attended an initial appointment and 24 review appointments were attended. The average number of days patients waited to attend an initial appointment was 148 days (range 31–308 days). Thirty patients (26 %) failed to attend. Self-reported reasons for non-attendance included that the patient: a) forgot the appointment (*n* = 8); b) did not receive an appointment letter (*n* = 3); c) no longer required the appointment (*n* = 7); d) rescheduled the appointment (*n* = 5), or; e) did not want to see a dietitian (*n* = 1). Six patients did not provide a reason for non-attendance.

**Fig. 1:**
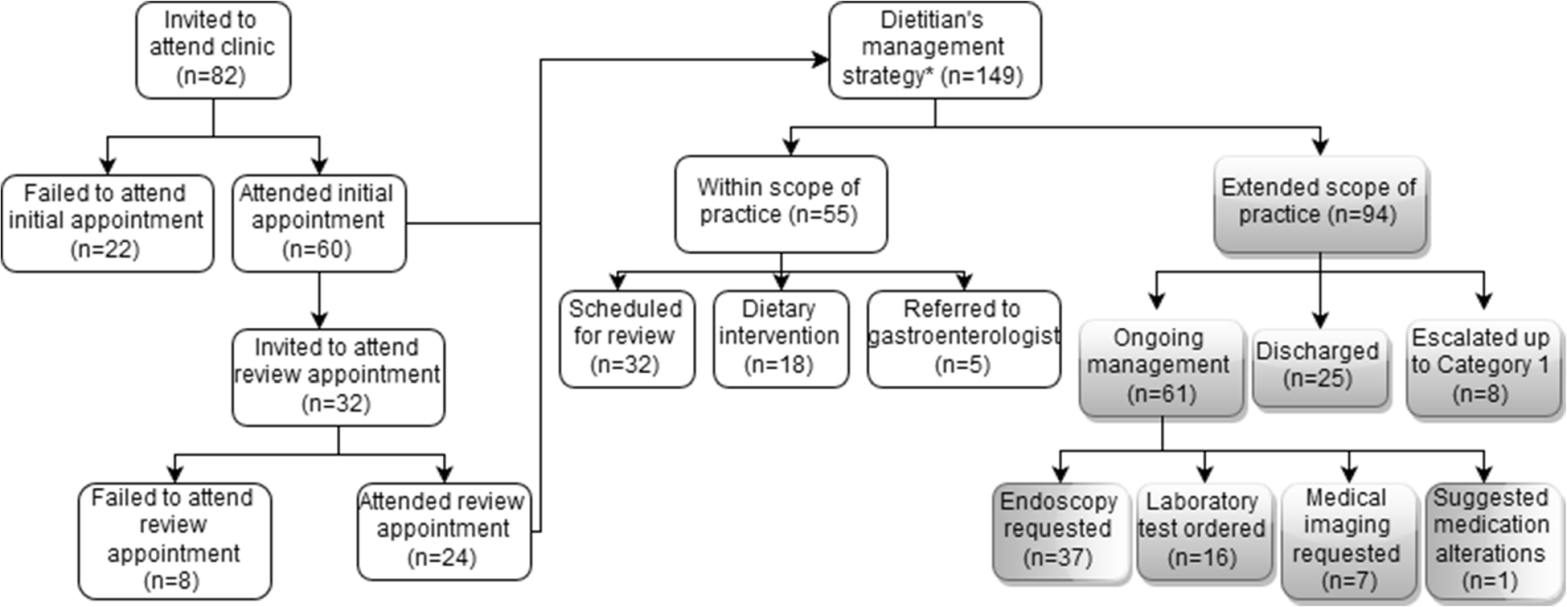
Management strategies for patients attending the dietitian-led gastroenterology clinic. * Some patients received more than one management strategy

A total of 149 management strategies were provided by the dietitian to patients, and 94 (63 %) of these involved the dietitian utilising an extended scope of practice (Fig. 1). Most patients (*n* = 47, 78 %) were managed exclusively by the dietitian, with approximately half of these (*n* = 25, 42 %) being discharged after dietetic management.

Information regarding patients’ presenting symptoms/conditions and outcomes after attending the clinic can be seen in Table 2. There was no significant difference between patient groups (i.e. lower GI, upper GI or other) for proportion of patients who received outcomes which involved the dietitian utilising an extended scope of practice.

**Table 2:** Patient outcomes by site of symptom/condition^a^

	Total (*n*)	Lower GI^b^	Upper GI^c^	Other^d^
Initial, attended				
- *n* (%)	60 (72)	37 (75)	30 (76)	5 (71)
- Age in years median (range)	51 (18–86)	51 (18–83)	50 (18–74)	61 (24–86)
Review, attended - *n* (%)	24 (75)	17 (74)	8 (72)	1 (50)
Extended scope implemented - *n* (%)	94 (63)	58 (85)	46 (68)	6 (75)

^a^Some patients presented with more than one condition/symptom and received more than one management outcome

The below n values refer to presenting conditions/symptoms in the initial consultation only

^b^Abdominal pain (*n* = 20), previous colonoscopy (*n* = 17), rectal bleed (*n* = 8), constipation (*n* = 7), diarrhoea (*n* = 5), bowel cancer (*n* = 2), altered bowel habits (*n* = 1), bloating (*n* = 1), irritable bowel syndrome (*n* = 1), food intolerance (*n* = 1)

^c^Gastroesophageal (*n* = 16), previous upper gastrointestinal endoscopy (*n* = 8), nausea/vomiting, (*n* = 5), dysphagia (*n* = 4), epigastric pain (*n* = 4), gastric ulcer (*n* = 3), upper gastrointestinal pain (*n* = 2), Barrett’s oesophagus (*n* = 2), oesophageal varices (*n* = 1), hiatus hernia (*n* = 1), hematemesis (*n* = 1), oesophageal stricture (*n* = 1)

^d^Anaemia (*n* = 1), high C-reactive protein (*n* = 1), high erythrocyte sedimentation rate (*n* = 1), high urea and electrolytes (*n* = 1), gall stones (*n* = 3), cholesterol polyps (*n* = 1), fatty liver (*n* = 1), biliary colic (*n* = 1

Forty patients (67 % of those who attended initial appointments) completed the patient satisfaction survey. All patients (100 %) agreed or strongly agreed that: a) the level of dietetic care and advice was adequate; b) they were satisfied with the outcome of the consultation, and; c) they were satisfied with the entire clinic experience. Only two respondents disagreed that they received access to health care faster via the clinic, while another two respondents disagreed that they had adequate information prior to attending. Four patients made comments on the survey that they did not receive an initial appointment letter, while another four indicated that they were unsure that the clinic allowed them access to health care earlier than expected.

## Discussion

This study found that patients had high levels of satisfaction with a dietitian-led gastroenterology clinic which operated under an extended scope of practice model. The dietitian utilising extended scope of practice under the supervision of the department’s gastroenterologist managed most patients without need for specialist assessment. This finding is similar to the results of a UK study, which found that at least one-third of all new gastroenterology referrals were appropriate for specialised dietetic advice [11].

To our knowledge, this is the first study to demonstrate the extended scope of practice model for a dietitian working in gastroenterology in the Australian context. Although examples of ESP dietitian roles exist in the literature, evidence regarding their effects is limited. Nevertheless, some small studies suggest that such roles are associated with similar positive outcomes to those observed in the present study. These include high levels of patient satisfaction, correct diagnosis, increased patient flow and decreased patient waiting times [10, 12–22]. Such studies also report a decreased workload for specialists, decreased costs to the health care system and increased satisfaction levels for health care professionals and dietitians themselves. Some of these outcomes were observed in the qualitative component of this study, which involved an exploration of various health care professionals’ perceptions of the dietitian-led clinic and extended scope model (Authors and published article reference blinded review (2015)).

High rates of inappropriate referrals puts increased pressure on gastroenterology services, further lengthening waiting lists [11]. Thus, better use of dietetic services may allow patients to be seen in a more timely manner. In the present study, the average waiting time for patients attending the dietitian-led clinic was 148 days, although there was a wide range of waiting time and no differentiation could be made between waiting periods for category 2 and category 3 patients. No national figures regarding patient waiting periods for gastroenterology outpatients are available. Nevertheless, state based figures published in July, 2015, indicate that 66 % of all category 1, category 2 and category 3 gastroenterology outpatients in Queensland were seen within the recommended time frames (30, 90 and 365 days respectively) [24]. Whether the clinic facilitated an improvement in patient flow is unclear. Hence, it is recommended that future studies investigate the degree to which dietitian-led extended scope clinics impact patient waiting times. Additionally, it would be beneficial to assess the entire patient journey (from initial consultation to patient discharge) in order to ascertain the true impact such clinics have on patients’ total time in the health system. For example, if there were significant waiting periods to receive endoscopies, reducing waiting times for the initial consultation may not necessarily reduce the length of time the patient spends in the health care system.

The patient satisfaction surveys indicated that patients were particularly satisfied with the level of support and care provided by the dietitian, their management outcomes after attending the clinic, and their overall clinic experience. Anonymous comments written on the surveys suggest that some patients did not receive a letter outlining the purpose of the clinic. This indicates that lack of funding and human resources (e.g. staff to be involved in the administrative aspects of the clinic) may be a barrier to achieving optimal levels of patient satisfaction. This is similar to the results of a UK study, which found that health professionals reported lack of resources to be a key barrier to establishing extended scope roles for allied health professionals [25]. Similar findings were reflected in the qualitative component of this study (Authors and reference of published article blinded for review (2015)). Furthermore, anonymous comments written on the patient satisfaction survey indicated that some respondents were unsure of how to answer the question that assessed their satisfaction with waiting periods to attend the clinic. This is possibly due to patients being unaware of the lengthy waiting times that are associated with attending public hospital outpatient departments.

In the UK, the extended scope of practice model is embedded into the health care system, and dietitians practising as ESPs perform their roles independent of the consultant. In this study, the gastroenterologist retained full jurisdiction over patient management, and the dietitian was required to gain approval before implementing any extended scope management strategies in order to minimise risk. This suggests that appropriate credentialing for ESP dietitians needs to be further investigated. Formalised training when developing allied health ESP positions will provide credibility to such roles, as well as ensure patient safety and practitioner accountability.

The findings of this study suggest that consultant dietitians in gastroenterology may be associated with various positive outcomes. These include decreased waiting periods, effective patient management, and heightened patient satisfaction levels. However, as this study only involved a sole dietitian, further investigation is recommended to assess whether an extended scope of practice for dietitians in gastroenterology and other areas of practice can improve outcomes for not only patients, but also the health system and health practitioners themselves. Additionally, this study was limited by a small sample size and the inability to link patient outcomes to the patient satisfaction surveys, thereby limiting the extent of data analysis that could be conducted. Furthermore, the present investigation failed to provide an assessment of costs associated with implementing the dietitian-led clinic, how the clinic effected patient waiting periods and differences in assessment, diagnosis and management strategies provided by the gastroenterologist and the dietitian. Hence it is recommended that future studies investigate these outcomes to further ascertain the logistics of implementing this model of health care.

Results of this study can be utilised to support the remodelling of health care systems, extending the roles of dietitians and other allied health professionals in specialist areas of clinical practice. This may potentially enhance outcomes for patients, practitioners and the health care system as a whole. Furthermore, this study suggests that remodelling the competency requirements and introducing more widespread credentialing and training opportunities to allow for dietetic specialisation may lead to more dietetic career pathways in the future, improving the outlook for the dietetic profession as a whole. Finally, outcomes of this study can be utilised by policy makers within governments and health departments who are pursing strategies to increase workforce productivity while optimising professional and patient outcomes.

## Conclusions

This study explored the extended scope model of care in the Australian setting, investigating the activities and outcomes of a dietitian acting with extended scope of practice in a gastroenterology outpatient clinical in a regional hospital in Queensland, Australia. Over three quarters of category 2 and 3 patients referred to the gastroenterologist could be managed exclusively in the dietitian-led clinic, with the rest requiring specialist input. Findings also suggest that this model of care resulted in decreasing patient waiting periods, increasing the effectiveness of patient management, and heightening patient satisfaction levels. Thus, the extended scope model of care could enhance the efficiency and acceptability of Australia’s public health system.

## Abbreviations

ESP: Extended scope practitioners
GI: Gastrointestinal
UK: United Kingdom
